# Comparison of Knowledge, Attitudes, and Practices of Educated and Uneducated Adults Regarding Human Immunodeficiency Virus in Karachi, Pakistan

**DOI:** 10.7759/cureus.1338

**Published:** 2017-06-12

**Authors:** Zainab Ahmad, Sara Sadiq, Mariam Asghar, Alizay Rashid Khan, Omer Arif, Syed Hamza Shah, Shahrukh Nadeem, Yamna Waseem, Rafi Aibani, Ammar salman Syed, Rabia M Mustafa, Zainab Abdulrahman, Kaneez Fatima

**Affiliations:** 1 Dow University of Health Sciences (DUHS), Karachi, Pakistan; 2 MBBS, Dow University of Health Sciences (DUHS), Karachi, Pakistan; 3 Department of Internal Medicine, Dow University of Health Sciences (DUHS), Karachi, Pakistan

**Keywords:** hiv, aids, education, attitude, practices, knowledge

## Abstract

**Background:**

Despite the high prevalence of human immunodeficiency virus (HIV) in Pakistan, no prior work has been done to specifically highlight the importance of education as a social vaccine against HIV. Therefore, our study focuses on differences in knowledge and practices regarding HIV and acquired immunodeficiency syndrome (AIDS) among educated and uneducated adults.

**Methodology:**

A cross-sectional study was carried out in which data was collected from all over Karachi. An individual was designated as educated if he had received education above primary school level. Individuals who had studied till primary school or less were considered uneducated. The questionnaire was split into four sections that assessed respondents' demographics, knowledge, attitudes, and practices regarding HIV/AIDS. Chi square test was used as the primary statistical test.

**Results:**

Out of the 446 adult participants, 235 (52.7%) were educated and 211 (47.3%) were uneducated. Educated participants were significantly more likely to have heard about HIV (183 vs. 39, p < 0.001) and had better knowledge about the symptoms of HIV/AIDS (p < 0.001). Among the participants who knew about AIDS, a greater percentage of uneducated (n = 28, 53%) than educated individuals (n = 68, 37%) believed that people suffering from AIDS should be isolated (p = o.o16) and that HIV can spread through water (40% vs 20% respectively, p < 0.001). Both educated (n = 49, 27%) and uneducated (n = 46, 89%) adults believed that awareness would help prevent the spread of HIV (p = 0.978) and were willing to educate their children about it (p = 0.696).

**Conclusion:**

Our study revealed a gap in the knowledge about HIV/AIDS between educated and uneducated adults. There is an urgent need for awareness programs that especially reach out to the uneducated masses that are otherwise uninformed about HIV and are under high risk of acquiring HIV.

## Introduction

Human immunodeficiency virus (HIV) is a deadly infection which leads to the subsequent development of acquired immunodeficiency syndrome (AIDS). HIV has remained a matter of concern for the last four decades and continues to threaten millions of lives globally. According to the Joint United Nations Programme on HIV/AIDS (UNAIDS), there are 36.7 million adults and children living with HIV/AIDS currently [1]. The highest number of patients has been reported in sub-Saharan Africa [1]. However, given its massive population density, around 2 million to 3.5 million HIV affected people reside in South Asia [2]. From the emergence of the first case in 1987 to the reporting an alarming toll of more than 100,000 HIV/AIDS cases [3], Pakistan is now considered a concentrated epidemic country. On average, around 3,600 people in the country die due to AIDS every year [4]. The HIV epidemic not only affects the health of individuals but also impacts households and communities and the development and economic growth of nations [5].​

Pakistan has a weak socio-economic background and also lags behind other lower-middle-income countries in non-income determinants of poverty, such as education and health [6]. Pakistan has a literacy rate of only 58% [7]. Many HIV/AIDS cases remain unreported and undiagnosed due to the social stigma attached to the infection, limited surveillance and voluntary counseling and testing systems, as well as the lack of knowledge among the general population and health practitioners [8]. Pakistan is a high risk country for HIV infections with low levels of condom use, a large population of long-distance truck drivers, a booming commercial sex industry, limited safety protocols for blood transfusions, high prevalence of sexually transmitted infections (STIs) with limited access to good-quality STI care, and increasing numbers of injection drug users (IDUs) [9].

Access and exposure to appropriate HIV/AIDS information and discussing it with others have the potential to impact knowledge, attitudes, beliefs, and sexual practices [10]. According to a study, alarming gaps in knowledge relating to HIV/AIDS were detected in young adults [11]. Considering the low literacy rate and increasing prevalence of HIV in Pakistan, the main objectives of this study were to compare and highlight the differences in HIV knowledge, attitudes, and practices between educated and uneducated adults. 

## Materials and methods

We conducted a cross-sectional population-based study after getting approval from the institutional Review Board of Dow University of Health Sciences. This study was conducted during February-March 2017. A person was designated as uneducated if they had no schooling or if they had only attended school till the primary school level; a person was recognized as educated if they had obtained education higher than the primary school level. As the focus of our study was adults, we made sure that only participants who were above the age of 20 years were included. Medical students, doctors, and health-related professionals were excluded to avoid bias due to professional knowledge. All the questionnaires that were not properly filled out, like the ones where more than one answer was marked for a question, were also excluded. An extensive questionnaire comprising 31 questions was designed and was divided into four sections. The first part collected information on the background of the participant, including their place of residence, age, gender, marital status, education, and socio-economic class. The following three parts focused on knowledge, attitudes, and practices. In the knowledge section, participants were asked about their knowledge of HIV/AIDS via a series of questions regarding the mode of transfer of HIV, the symptoms of HIV, and if AIDS can be treated and cured. In the attitude section, they were asked if HIV is a public health problem and if they believe that it should be discussed publicly to increase awareness. Additionally, a few questions regarding the attitude of people towards HIV-infected people were asked. In the final section, individuals were asked about their practices towards HIV and towards patients with HIV. They were asked if they would educate other people and their own children to help spread awareness and thus prevent its spread, and whether they would take certain precautions against HIV. Before carrying out the study, the questionnaire was approved by an infectious disease specialist with expertise in HIV, and a pilot study was carried out to check the questionnaire's relevance. We included 235 educated and 211 uneducated participants to have an approximately equal number so we could have a fair comparison.

Data were collected by interviewers, all of whom had been given prior instructions and training on how to carry out the survey, so as to eliminate any interviewer bias. People were approached at random in different places in Karachi. They were first briefed about where we were from and the aim of our study. We approached 473 individuals, of whom 446 individuals agreed while the remaining 27 refused; hence, the cooperation rate was 94.3%. Participants who had issues with questions related to sexual intercourse refused to continue. Written consent was taken from every individual before moving onto the questionnaire. Educated people were handed questionnaires to fill them out themselves, and any assistance needed in understanding the questions was provided by the principal investigator. English is not the native language of Pakistan; hence, for those who did not know English, especially the uneducated participants, the questionnaire was translated into Urdu and questions were explained and asked verbally. The interviewer ensured that the questionnaire was completely filled, and if not, then the unanswered questions were assumed to be due to a lack of knowledge, which provided us with the percentage of people who were not fully aware about HIV/AIDS. The participants involved in the study were assured that the data collected would be kept anonymous and no personal questions were asked other than those required for the research.

Once all the data were collected, it was compiled and entered into Statistical Package for Social Sciences (SPSS) software version 23.0. For categorical variables, frequencies and percentages have been reported, while for continuous variables, mean and standard deviations have been reported. Each question was analyzed individually with respect to demographic variables such as age, education, marital status, and social class through the Chi square test.

## Results

The study included 235 educated and 211 uneducated participants. Out of the total 446 participants, only 222 (49.8%) had heard about HIV. Among those who had heard about HIV, 183 (82%) were educated and 39 (18%) were uneducated. When asked about AIDS, 264 participants were aware of it; 191 (72%) educated and 73 (28%) uneducated (Table 1).

**Table 1:** Distribution of knowledge between educated and uneducated individuals

		Do you know about AIDS			
		Yes (n = 264)		No (n = 182)	
		Educated	Uneducated	Educated	Uneducated
Have you heard about HIV	Yes (n = 222)	156	31	27	8
	No (n = 224)	35	42	17	130

**Figure 1:**
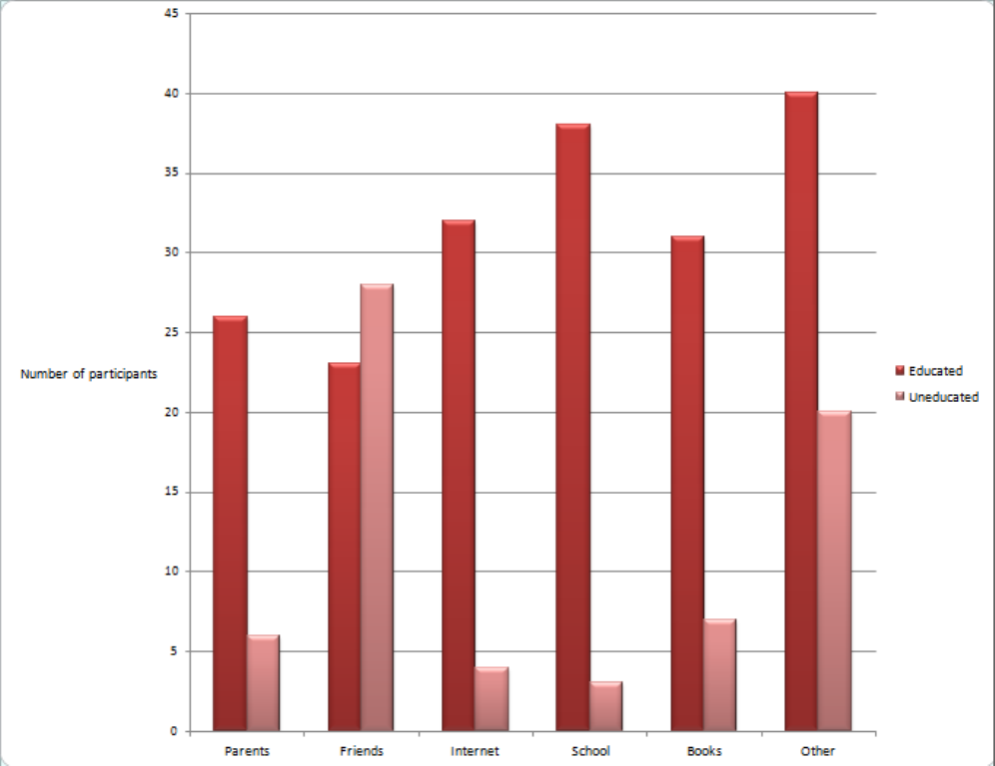
Source of knowledge among people who know about AIDS

Almost a similar number of men and women knew about AIDS (125 vs 139 respectively) (p = 0.115) and had heard about HIV (110 vs 112 respectively) (p = 0.705). A greater percentage of unmarried people knew about AIDS (n = 104, 72.7%) compared to those who were married (n = 158, 52.7%) (p < 0.001). The sources of knowledge related to AIDS, among educated and uneducated participants, is shown in Figure 1.

### Knowledge

Among the 446 participants included in the survey, 187 knew about AIDS and HIV both. Among those 187, 31 (17%) were uneducated and 156 (83%) were educated. 166 of them agreed that AIDS is caused by HIV. Almost similar percentages of educated (n = 109, 77%) and uneducated people (n = 16, 76%) people said that AIDS could be delayed in a person infected with HIV. 168 individuals said that it is important to get tested for HIV, especially before marriage, and 28 of them were themselves tested (Table 2). There was no significant relation between education status and having been tested for HIV (p = 0.609).

**Table 2:** Differences between people who know about both, HIV and AIDS

		People who know about HIV and AIDS both (n=187)		
		Education		Total
		Uneducated (n=31, 17%)	Educated (n=156, 83%)	
Does HIV cause AIDS	Yes	25 (93%)	141 (94%)	166
	No	2 (7%)	9 (6%)	11
	Total	27	150	
Can AIDS be delayed in a person with HIV	Yes	16 (76%)	109 (77%)	125
	No	5 (24%)	33 (23%)	38
	Total	21	142	
Do you think it is important to get tested for HIV	Yes	23	145	168
	No	3	9	12
	Total	26	154	
Tested for HIV	Yes	7 (25%)	21 (14%)	28
	No	21 (75%)	134 (86%)	155
	Total	28	155	

Out of the 446 participating adults, 264 knew about AIDS; however, uneducated participants had lesser knowledge about the symptoms of AIDS (p < 0.001). Educational standing did not significantly influence knowledge regarding the presence of a cure for the illness, as 93 (50%) educated and 38 (61%) uneducated participants thought AIDS was curable (p = 0.310). The majority of both educated and uneducated participants (73% and 70%, respectively) believed that HIV is not well documented within Pakistan (Table 3). 

**Table 3:** Differences of knowledge in educated and uneducated individuals

		People who know about AIDS (n=264)		P - values
		Uneducated (n=73)	Educated (n=191)	
Is HIV well documented within Pakistan	Yes	19 (30%)	49 (27%)	0.256
	No	44 (70%)	134 (73%)	
Can AIDS be cured	Yes	38 (61%)	93 (50%)	0.310
	No	24 (39%)	94 (50%)	
Do you know about symptoms of AIDS	Yes	12 (18%)	104 (55%)	< 0.001
	No	56 (82%)	87 (45%)	

### Attitude and Practices

Among the people who knew about AIDS (n = 264), a majority of educated (n = 170, 92%) and uneducated (n = 46, 89%) people believed that awareness could help prevent AIDS and said that they would educate their children about AIDS. Education did not significantly affect the popular belief that AIDS is a public health problem in Pakistan (p = 0.168). Great numbers of both, educated (n = 177) and uneducated (n = 50) adults believed that the spread of HIV could be prevented if protection was used during sexual intercourse (p = 0.117). A greater percentage of uneducated (53%) than educated (37%) participants believed that people suffering from AIDS should be isolated. When asked whether those same people should be permitted to use public washrooms, more educated (n = 76, 42%) than uneducated (n = 18, 35%) individuals replied negatively (Table 4).

**Table 4:** Differences of attitude and practices between educated and uneducated individuals

		People who know about AIDS (n=264)		P - values
		Uneducated (n=73)	Educated (n=191)	
Can awareness help prevent AIDS	Yes	46 (89%)	170 (92%)	0.978
	No	6 (11%)	15 (8%)	
Would you educate your children about AIDS	Yes	48 (89%)	171 (92%)	0.696
	No	6 (11%)	14 (8%)	
Is AIDS a public health problem	Yes	46 (73%)	155 (83%)	0.168
	No	17 (27%)	33 (17%)	
Should AIDS patients be isolated	Yes	28 (53%)	68 (37%)	0.016
	No	25 (47%)	114 (63%)	
Should AIDS patients be allowed to use public washrooms	Yes	33 (65%)	105 (58%)	0.013
	No	18 (35%)	76 (42%)	
Can spread of HIV be avoided by using protection during intercourse	Yes	50 (89%)	177 (96%)	0.117
	No	6 (11%)	8 (4%)	

An overwhelming majority of both, educated (95%) and uneducated (86%) adults, thought that HIV can be spread by using already used intravenous needles. Likewise, the idea that HIV spreads through direct blood contact between infected and uninfected individuals was also a famous belief among educated and uneducated participants (92% and 80%, respectively). More educated than uneducated people said that HIV can pass from a mother to the child through breast milk (p = 0.02) and that it could not be spread by being around an infected person (p < 0.001), or by his sneezing and coughing (p < 0.001). Significantly a higher percentage of uneducated individuals believed that HIV can spread through water (p < 0.001) and through mosquito bites (p < 0.001) (Table 5).

**Table 5:** Knowledge about presupposed factors that may help spread HIV

Factors		People who know about AIDS (n=264)		P - values
		Uneducated (n=73)	Educated (n=191)	
IV needles	Yes	47 (86%)	176 (95%)	0.088
	No	8 (14%)	9 (5%)	
Direct blood contact	Yes	44 (80%)	170 (92%)	0.207
	No	11 (20%)	15 (8%)	
Breast milk	Yes	20 (39%)	102 (56%)	0.020
	No	32 (61%)	81 (44%)	
Sneeze and cough	Yes	26 (51%)	39 (22%)	< 0.001
	No	25 (49%)	142 (78%)	
Touching/being around patient	Yes	18 (35%)	28 (15%)	< 0.001
	No	34 (65%)	155 (85%)	
Water	Yes	23 (40%)	37 (20%)	< 0.001
	No	28 (60%)	149 (80%)	
Mosquito bite	Yes	29 (56%)	56 (31%)	< 0.001
	No	23 (44%)	126 (69%)	

## Discussion

This study shows the gap in the knowledge regarding HIV and AIDS between the educated and the uneducated populations. 72% of the educated participants knew about AIDS and 82% had heard about HIV. In addition, 28% of the uneducated participants knew about AIDS and 18% had heard about HIV. Consistent with prior findings in India, our study exhibits a positive relationship between information and education regarding HIV [12]. Literate residents have a tendency to have better self-viability, mindfulness, and greater adherence to sound practices that are essential for HIV avoidance. They are also more capable of translating and adapting to the information provided by peers, television, other media and advertisements, which was also seen in the UNAIDS review of research [13]. A recent demographic survey showed that education is positively related to knowledge of HIV, which underpins the discoveries in this review [14].

The first case of HIV in Pakistan was reported in 1897 [15], and since then the prevalence of the infection has steeply increased. A majority of the people answered that it was important to get tested for HIV, especially before marriage, but surprisingly, the study found that while they do think it is important to get tested, a majority of them were not tested for HIV themselves. One of the fundamental reasons participants do not get tested for HIV is that they are terrified. According to a prior study [16], some demonstrated essential feelings of dread of needles, and of the test itself. Beyond the fundamental dread of the test itself, some showed a fear of the shame related with HIV. Secondly, since Pakistan’s most commonly practiced religion condemns pre-marital or extra-marital sex and is also against homosexuality, they believe this prevents the spread of HIV; individuals have the false impression that Pakistan is sheltered from its spread and so would prefer not to get tested [17]. Another reason is the cost of the test, as cited by a couple of the participants. This is a noteworthy reason amongst the uneducated populace, who are more likely to be unemployed or have lower wages, and hence hold a weak financial foundation. The individuals who got their HIV test done were mostly found to have done it as part of some other treatment.

The study also highlighted some misconceptions that people have regarding HIV. Firstly, over half of the people who knew about AIDS thought that it was curable. They trust that medication at this point ought to have sufficiently advanced to discover a cure. A substantial proportion of the uneducated population was incognizant of the fact that HIV can spread through breast milk. This emphasizes the need for awareness regarding the various modes of transmission, especially amongst expecting females. About 82% of uneducated and 45% of educated participants had no clue about the symptoms of HIV, which further reflects the lack of knowledge of this disease. A large share of the respondents had correct information regarding the modes of transmission. However, it was discovered that a number of participants believed that HIV is likewise transmitted through mosquito bites, water, sneezing and coughing, and by casual contact, all of which were also reported in a study of Belgian university students [18]. The best technique for preventing the spread of HIV, other than forbearance from sex, is the use of condoms [19]. In our study, the majority of the adults responded that they agree with the use of protection as a method for safe sexual intercourse. This is in contrast with the general mindset of the population, who think that the use of a condom is either not allowed in Pakistan’s common religion or makes sexual intercourse "less exciting" [20]. A general feeling of sympathy resides within the respondents for HIV-positive people, as they believe that infected patients should have access to public washrooms and should not be isolated.

The survey indicates that a general increase in the literacy rate results in more awareness and knowledge of AIDS; therefore, any attempts to improve and spread education will be beneficial. Public interest is shown in the results, as a vast majority of participants believe in awareness and educating their children about AIDS. Past studies reveal that an emphasis on raising awareness with respect to HIV is integral to all HIV counteractive action techniques [21]. Individuals ought to be instructed through radio, TV, and daily papers. Classes and wellbeing-related workshops about the significance of hygiene and about the modes of transmission of HIV should be held. Campaigns should be carried out in less privileged areas to target uneducated people belonging to the lower class. Like other developed countries, sexual health care centers should be set up in Pakistan. Contraceptives should be further promoted. These centers should work alongside legislative and non-government associations in the red territories of Pakistan, where the rate of AIDS casualties are more. The stigma regarding HIV should also be addressed and individuals should be taught that HIV patients ought not to be secluded or socially disgraced; this will encourage more individuals to take HIV tests and will promote open familiarity with HIV. It is also essential that young children learn about the syndrome through reliable sources as misinformed or underinformed sources can create false perceptions in their minds.

Steps to improve this situation have been taken by over 50 non-governmental organizations (NGOs) in Pakistan who not only have outreach schemes such as needle-exchange programs for intravenous drug users (IDUs), but also programs to spread awareness about HIV to the general public. A worthwhile mention is 'AMAL', which means 'action' in Pakistan's national language, Urdu. It has outreach HIV training programs not only for IDUs, but also for the out-of-the-limelight population.

In spite of the fact that this article lays the groundwork for future research regarding this matter in Pakistan, there are a few limitations that need to be considered. Firstly, the sample size was small; therefore, it should be noted that the data are applicable only to the population studied. Secondly, the number of educated and uneducated adults was slightly unequal. Moreover, the questionnaires were filled just by the individuals that consented to answer it, which suggests that respondents were mindful of HIV in contrast to the individuals who declined to respond. 

## Conclusions

The findings of this study conclude that people have a low level of education about HIV/AIDS. Common practices are not followed and an increase in awareness has to be implemented, possibly by means of various media, if the epidemic is to be checked within the populace of Pakistan. Preventive medical programs on HIV/AIDS should also be designed according to the stratum of educational qualification to which one belongs. Efforts should likewise be made to educate people that a discussion about HIV/AIDS publicly must not be considered taboo.
